# Supplementary material for: Cryo-EM analysis of V/A-ATPase intermediates reveals the transition of the ground-state structure to steady-state structures by sequential ATP binding
Source: J Biol Chem. 2023 Jan 7;299(2):102884. doi: 10.1016/j.jbc.2023.102884 (PMC9971907; doi:10.1016/j.jbc.2023.102884)
Supplement: Supporting information [file mmc1.pdf]

Supporting information to:

Structural transition of the ground-state structure to steady-state structures by sequential binding of ATP to V/A-ATPase

Atsuko Nakanishi, Jun-ichi Kishikawa, Kaoru Mitsuoka, and Ken Yokoyama

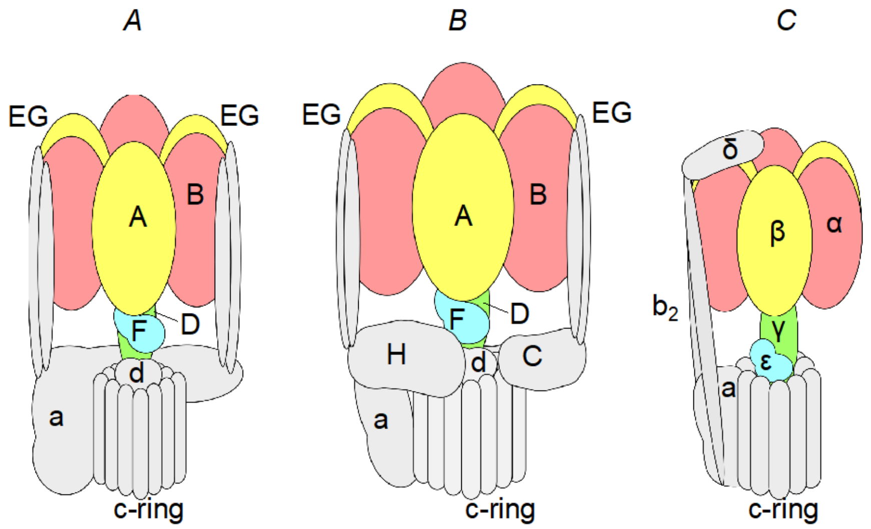

Figure S1. Schematic representation of rotary ATPases.

Schematic of the subunit composition of different types of rotary ATPases: prokaryotic V/A-ATPase (**A**), eukaryotic V-ATPase (**B**), and prokaryotic F-ATPase (**C**). The hydrophilic V_1/F_1 domain is represented by various colors, and the hydrophobic V_o/F_o domain is colored in gray.

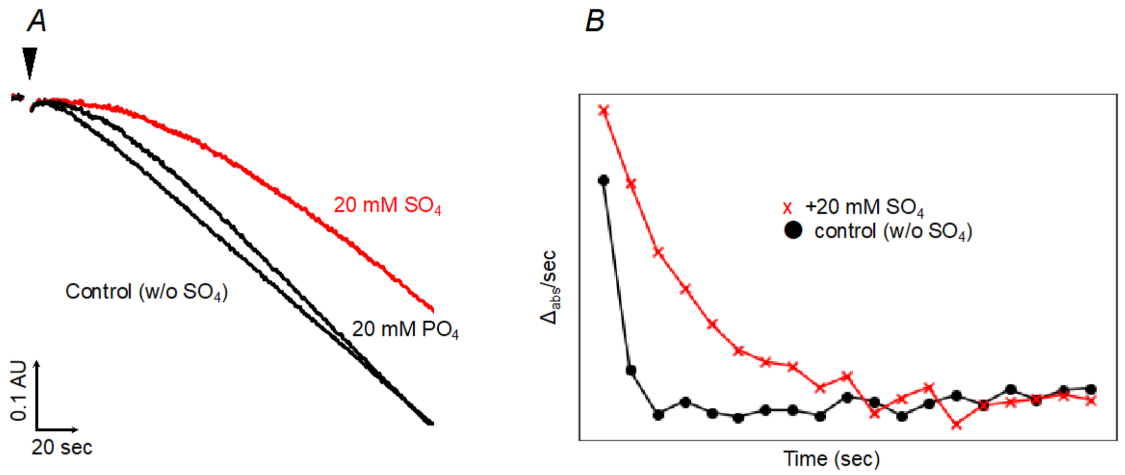

Figure S2. Sulfate effect on ATP hydrolysis by the V/A-ATPase. A, Time-course of ATP hydrolysis catalyzed by the nucleotide-free V/A-ATPase ($V_{nucfree}$) under saturating ATP (a final concentration of 4mM ATP) condition in the absence (Control) or presence of sulfate (20 mM SO_4) as indicated. There were almost no changes in ATPase activity due to adding phosphate (20 mM PO_4). **B**, Plots show the differential of the ATPase activity per unit time (20 s). It takes even more time to reach steady state in the presence of sulfate (red) in the absence of sulfate (black).

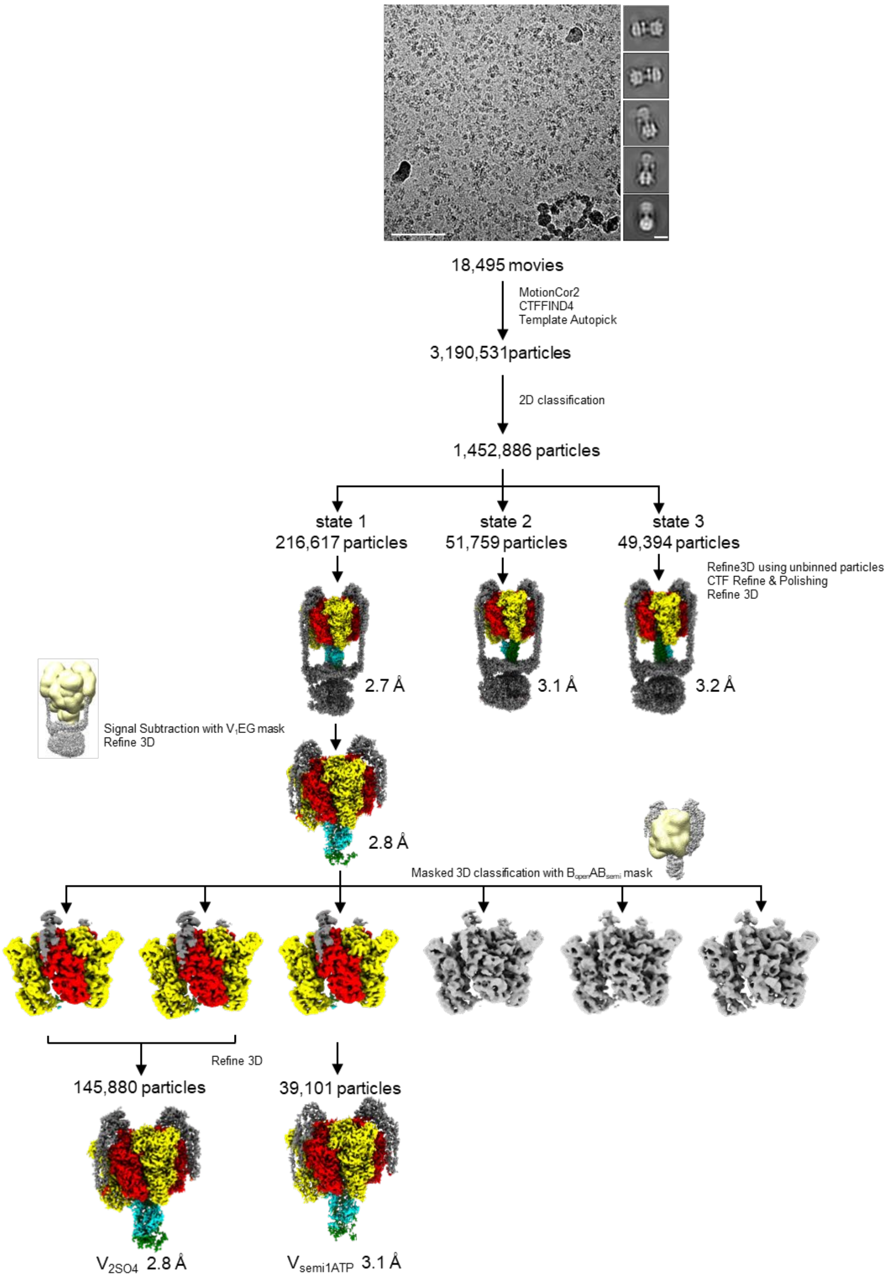

Figure S3. Cryo-EM processing workflow of the V/A-ATPase activated by 20 µM ATP for 60 s. A processing pipeline for 3D reconstruction of the nucleotide-free V/A-ATPase ($V_{nucfree}$) under much lower ATP concentration than K_m value (a final concentration of 50 µM) with 20 mM sulfate. Example micrograph (top left, scale bar; 100 nm), 2D class average images (top right, scale bar 100 Å), and cryo-EM data analysis workflow (bottom) are shown. Masked 3D classification allowed us to obtain two other maps with improved quality of the V_1EG region at 2.8 Å (V_{2SO4}) and 3.1 Å ($V_{semi1ATP}$), respectively.

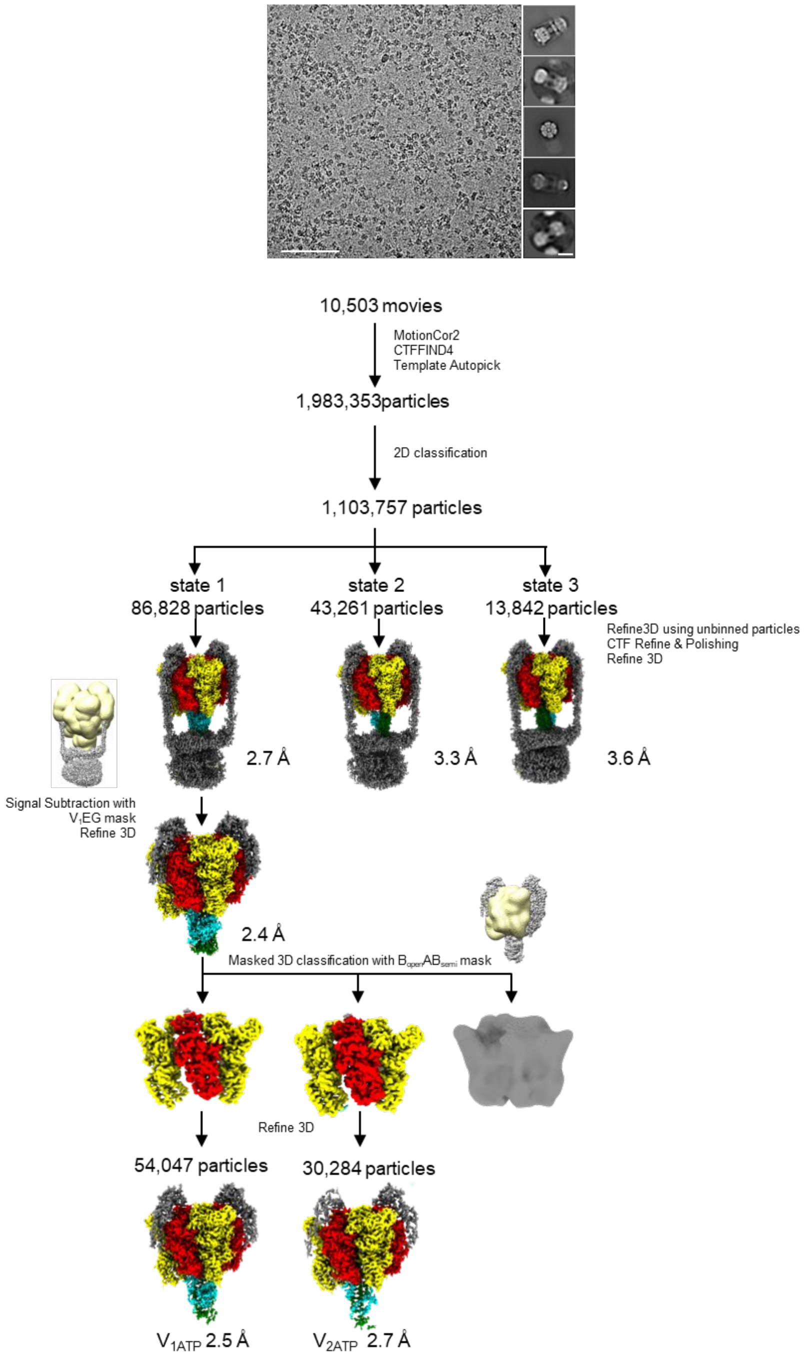

Figure S4. Cryo-EM processing workflow of the V/A-ATPase activated by 4 mM ATP for 5 s. A processing pipeline for 3D reconstruction of the nucleotide-free V/A-ATPase ($V_{nucfree}$) under saturating ATP concentration with 20 mM sulfate. Example micrograph (top left, scale bar; 100 nm), 2D class average images (top right, scale bar 100 Å), and cryo-EM data analysis workflow (bottom) are shown. Masked 3D classification allowed us to obtain two other maps with improved quality of the V_1EG region at 2.5 Å (V_{1ATP}) and 2.7 Å (V_{2ATP}), respectively.

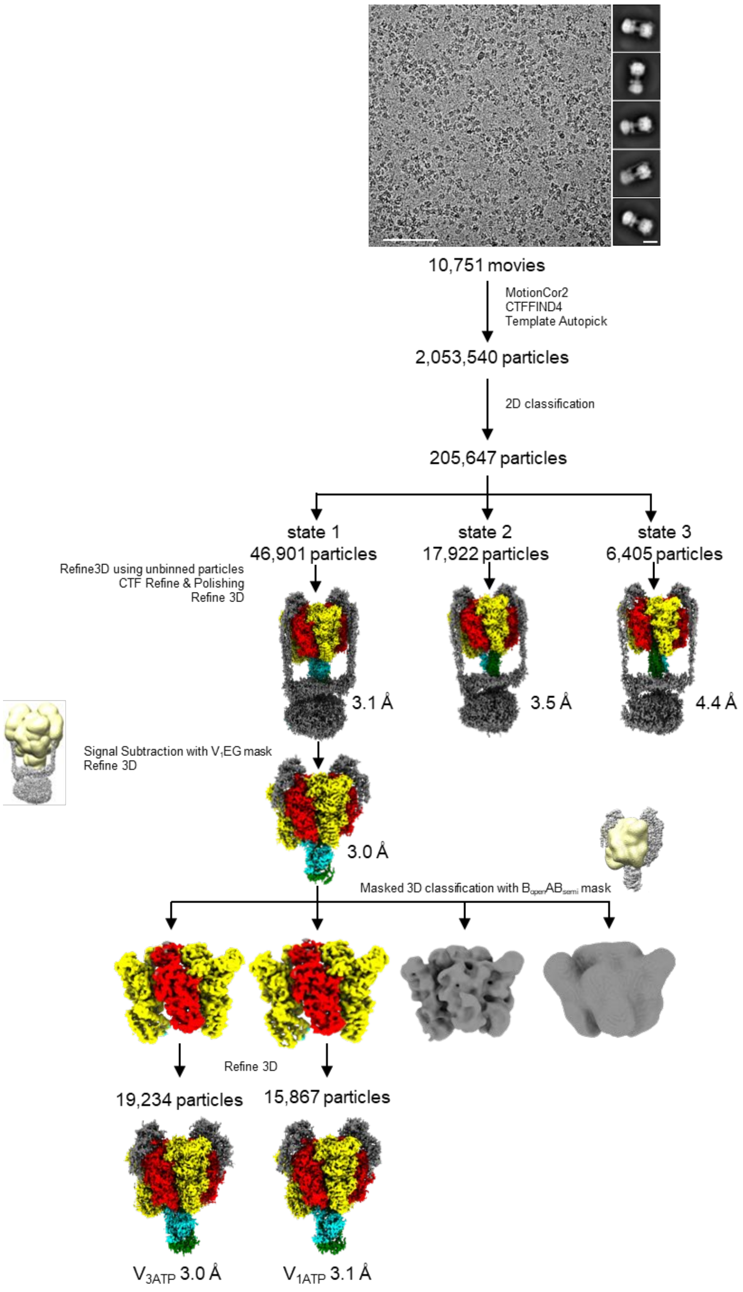

Figure S5. Cryo-EM processing workflow of the V/A-ATPase activated by 4 mM ATP for 30 s. A processing pipeline for 3D reconstruction of the nucleotide-free V/A-ATPase ($V_{nucfree}$) under saturating ATP concentration with 20 mM sulfate. Example micrograph (top left, scale bar; 100 nm), 2D class average images (top right, scale bar 100 Å), and cryo-EM data analysis workflow (bottom) are shown. Masked 3D classification allowed us to obtain two other maps with improved quality of the V_1EG region at 3.0 Å (V_{3ATP}) and 3.1 Å (V_{1ATP}), respectively. The V_{1ATP} map was identical to the V_{1ATP} map obtained from the dataset of 5 sec reaction (Figure 3-figure supplement 2); hence, a higher resolution map of V_{1ATP} was used for further analysis.

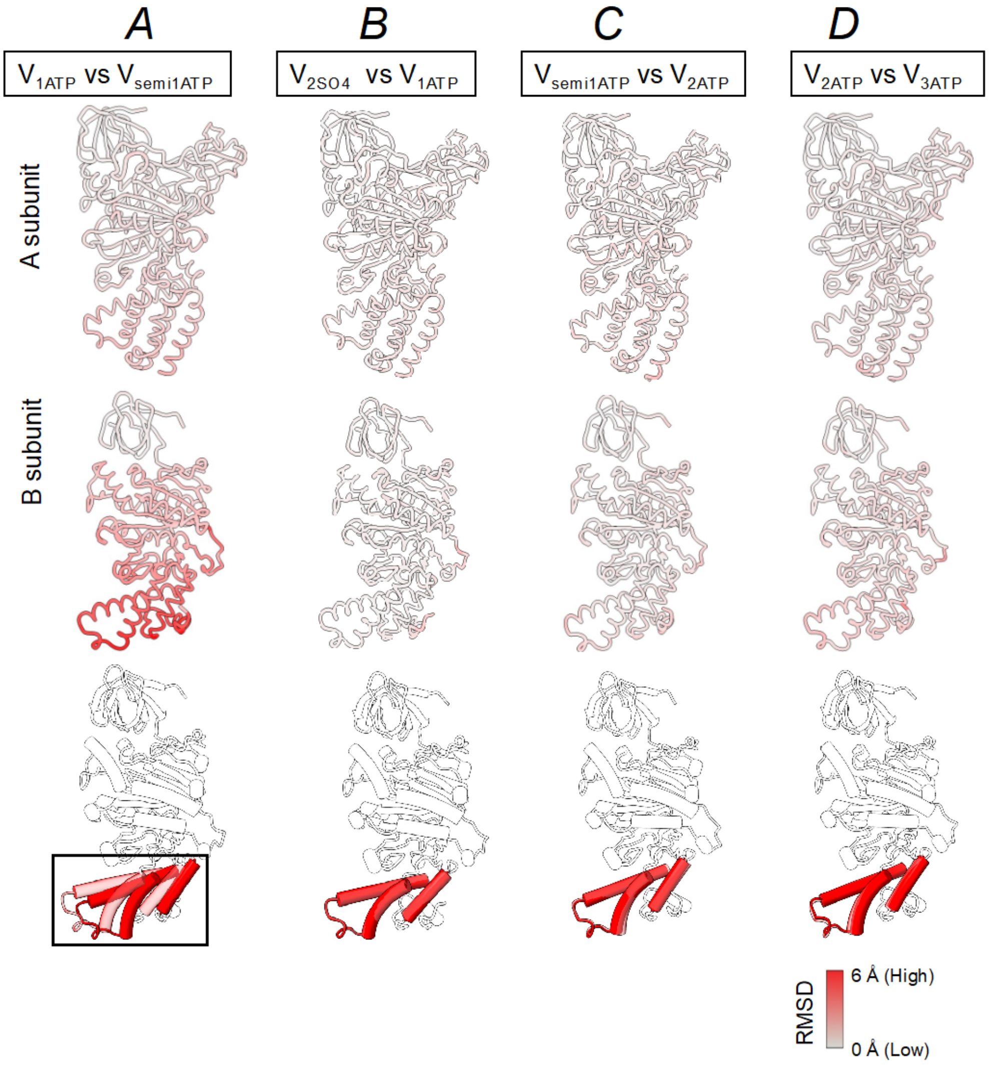

Figure S6. Comparison between intermediates in each semi-closed A and B subunits. The semi-closed A and B subunits of each intermediate were superimposed on the β barrel domain (A: 1–70 a.a. and B: 1–70 a.a., respectively). Models are colored by C_α root mean square displacement (RMSD) values calculated using C_α atoms; gray (small changes) to red (large changes). C_α RMSD values between V_{1ATP} and $V_{semi1ATP}$ **(A)**, V_{2SO4} and V_{1ATP} **(B)**, $V_{semi1ATP}$ and V_{2ATP} **(C)**, V_{2ATP} and V_{3ATP} **(D)** are shown in top. Comparisons of the C-terminal helix bundle of B subunit of V_{1ATP} (transparent) and $V_{semi1ATP}$ (opaque) (**A**), V_{2ATP} (transparent) and V_{1ATP} (opaque) (**B**), $V_{semi1ATP}$ (transparent) and V_{2ATP} (opaque) (**C**), V_{2ATP} (transparent) and V_{3ATP} (opaque) (**D**) are shown in bottom. The long α-helix of the C-terminal helix bundle of the B subunit are shown as cylinders to clarify the structural differences.

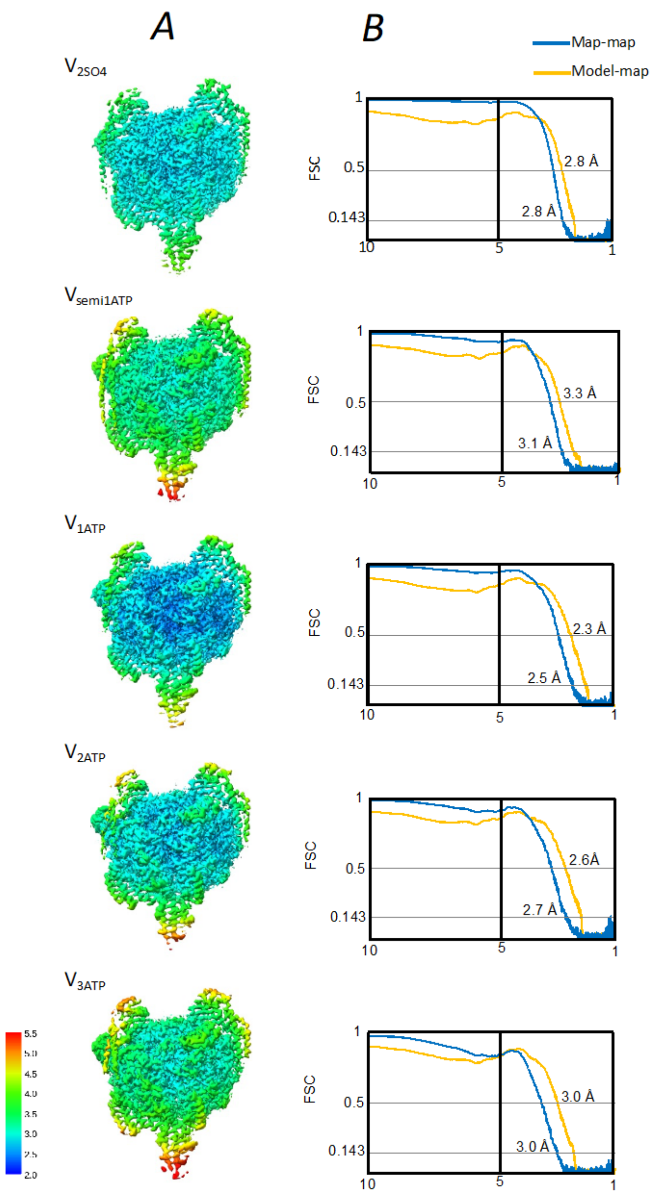

Figure S7. Cryo-EM map validation for the V_1EG domain of the intermediates. A, Local resolution of the final maps of initial intermediates as indicated. Resolutions are color-coded by scale bars. **B**, Fourier shell correlation (FSC) curves of reconstructed map of V_1EG domain.

A/444-573	V_{2SO4}	$V_{semi1ATP}$	V_{1ATP}	V_{2ATP}	V_{3ATP}
V_{2SO4}		0.898	0.38	1.294	1.917
$V_{semi1ATP}$	0.898		**1.092**	0.541	1.143
V_{1ATP}	0.38	**1.092**		1.465	2.083
V_{2ATP}	1.294	0.541	1.465		0.761
V_{3ATP}	1.917	1.143	2.083	0.761	

B/374-446	V_{2SO4}	$V_{semi1ATP}$	V_{1ATP}	V_{2ATP}	V_{3ATP}
V_{2SO4}		5.11	0.24	4.813	5.223
$V_{semi1ATP}$	5.11		**5.225**	0.667	0.778
V_{1ATP}	0.24	**5.225**		4.923	5.321
V_{2ATP}	4.813	0.667	4.923		1.05
V_{3ATP}	5.223	0.778	5.321	1.05	

Table S1. RMSD values for C-terminal domain of semi-closed A (top) and B (bottom) subunit. Subunits were superimposed on the β-barrel domain (1–70 a.a), and then C_α root means square displacement (RMSD) values for the backbone (444–573 a.a for A subunit, 374–446 a.a for B subunit) were calculated. The values for $V_{semi1ATP}$ versus V_{1ATP} are indicated in bold.

	V_{2SO4} **EMD-34365 PDB ID 8GXY**	$V_{semi1ATP}$ **EMD-34366 PDB ID 8GXZ**	V_{1ATP} **EMD-34362 PDB ID 8GXU**	V_{2ATP} **EMD-34363 PDB ID 8GXW**	V_{3ATP} **EMD-34364 PDB ID 8GXX**
**Data collection and processing**					
Magnification	60000	60000	105000	105000	105000
Voltage (kV)	300	300	300	300	300
Electron exposure ($e^-/Å^2$)	50	50	50	50	50
Defocus range (μm)	-1.2 to -2.4	-1.2 to -2.4	-0.8 to -1.8	-0.8 to -1.8	-0.8 to -1.8
Pixel size (Å)	0.81	0.81	0.83	0.83	0.83
Symmetry imposed	C1	C1	C1	C1	C1
Initial particle images (no.) (after 2D)	1452886		1103757		205647
Final particle images (no.)	145880	39101	54047	30284	19234
Map resolution (Å)	2.8	3.1	2.5	2.7	3
FSC threshold	0.143	0.143	0.143	0.143	0.143
**Refinement**					
Initial model used (PDB accession no.)	This study	This study	This study	This study	This study
Model resolution (Å)	2.8	3.3	2.3	2.6	3
FSC threshold	0.5	0.5	0.5	0.5	0.5
Map-sharpening B factor ($Å^2$)	-76	-67	-41	-39	-67
Model composition					
Nonhydrogen atoms	29472	29499	29503	29530	29553
Protein residues	3788	3788	3788	3788	3788
Ligands	SO4: 2	SO4: 1, ATP: 1	SO4: 2, ATP: 1	SO4: 1, ATP: 2	ATP: 3
B factors ($Å^2$)					
Protein	58.51	91.33	53.2	70.31	65.86
Ligand	58.35	115.67	73.07	74.12	63.31
R.m.s. deviations					
Bond lengths (Å)	0.005	0.006	0.007	0.006	0.006
Bond angles (°)	0.519	0.535	0.574	0.546	0.557
**Validation**					
MolProbity score	1.3	1.66	1.44	1.36	1.49
Clashscore	5.16	6.61	4.64	5.14	5.57
Rotamer outliers (%)	0	0	0	0	0.06
Ramachandran plot					
Favored (%)	97.87	95.72	96.76	97.61	96.92
Allowed (%)	2.1	4.25	3.24	2.36	3.06
Disallowed (%)	0.03	0.03	0	0.03	0.03

Table S2. Cryo-EM data collection, refinement, and validation statistics.
